# Malignant Transformation of Actinic Cheilitis: A Decade-long Retrospective Study in Southern Brazil

**DOI:** 10.4317/jced.61590

**Published:** 2024-06-01

**Authors:** Ely G. Pierin, Laurindo M. Sassi, Juliana L. Schussel

**Affiliations:** 1 Post-graduation Program in Dentistry, Department of Stomatology, Universidade Federal do Paraná; 2 DDS MSc PhD. Department of Oral and Maxillofacial Surgery, Erasto Gaerner Cancer Center; 3 DDS MSc PhD. Post-graduation Program in Dentistry, Department of Stomatology, Universidade Federal do Paraná. Department of Oral and Maxillofacial Surgery, Erasto Gaerner Cancer Center

## Abstract

**Background:**

Actinic cheilitis (AC), an oral potentially malignant oral disorder (OPMD), predominantly affects fair-skinned individuals, particularly males, with a higher prevalence in their sixth and seventh decades. In the Southeast region of Brazil, oral cancer ranks as the fourth most common tumor among males, with squamous cell carcinoma (SCC) constituting 90% to 95% of lip tumor cases, primarily impacting the lower lip. This retrospective study aims to evaluate the malignant transformation rate in previously diagnosed AC patients between 2008 and 2018, utilizing biopsy records.

**Material and Methods:**

We retrospectively reviewed medical records of lip lesion patients at the Department of Oral and Maxillofacial Surgery (DOMS) during the stated period, collecting demographic and medical data for analysis.

**Results:**

Among the 224 analyzed AC cases, 67.8% were male, with an average age of 65 years. Approximately 87.6% of patients reported occupational exposure to AC-associated risk factors. Malignant transformations occurred in 27 patients (12.05%).

**Conclusions:**

Our study underscores the possible effect of early intervention and preventive measures in stabilizing AC lesions and averting their progression to malignancy. These findings underscore the significance of prompt AC diagnosis and management to mitigate the risk of malignant transformation.

** Key words:**Actinic cheilitis, oral squamous cell carcinoma, malignant transformation.

## Introduction

Actinic cheilitis (AC) is a potentially malignant oral disorder that predominantly affects fair-skinned males. It is most prevalent during the sixth and seventh decades of life and is closely associated with occupational risks and exposure to ultraviolet (UV) radiation. AC primarily affects the lower lip and is commonly observed in individuals with excessive sunlight exposure, such as fishermen, farmers, and surfers, putting them at risk of progressing to lip cancer (1,2). The main cause of AC is chronic and cumulative exposure to ultraviolet radiation (UV) (3,4). Additionally, it may be linked to immunosuppressed patients and individuals with certain genetic susceptibilities (2,5). In the acute form of AC, characteristic symptoms include dryness, edema, redness, which can advance to vesicles, blisters, crusts and ulcerations (6). Removal of the etiological factor leads to a lesion regression. On the other hand, the chronic form presents as an elevated lower lip, often extending to the commissure. These diffuse, asymptomatic lesions cause atrophy of the red lip border, erasing the margin between the red and skin regions of the lower lip. They exhibit color changes with erythroleukoplastic areas, lose elasticity, and may cause erosion in some cases. One common clinical finding in AC is the loss of demarcation between the lip and skin boundaries, which can be mistaken for signs of aging, potentially leading to delayed medical attention (4).

Actinic cheilitis can be classified into four grades based on distinct clinical characteristics that complement each other as the condition progresses. Grade I present with dryness, flaking and erythema on the lips. In Grade II, there is vermillion lip atrophy with pale areas and thin surface, and effacement between the mucosa and skin boundary, along with lip swelling. Grade III involves rough and scaly areas on the vermillion lip, hyperkeratotic regions, and some firmness upon palpation. In Grade IV, erosion or ulceration is found in one or more vermilion lip sites, possibly accompanied by leukoplakia in more traumatic areas and a history of cigarette consumption. These lesions indicate an ongoing malignancy process, especially if they are accompanied by firm areas upon palpation (6). The grading system helps healthcare professionals identify the severity of AC, aiding in appropriate management and intervention strategies.

Histologically, actinic cheilitis is characterized by epithelial hyperkeratosis, hyperplasia, and/or atrophy. As the condition advances, dysplasia emerges as a significant histological feature. Remarkably, it has been reported that 100% of AC cases exhibit some degree of dysplasia, coupled with solar elastosis, inflammation, and vasodilation (4) . Due to these attributes, AC is classified as an oral potential malignant disorder, marked by unpredictable progression regardless of histological grading (5). Carcinogenesis typically stems from gradual exacerbation of epithelial dysplasia, often perpetuated by continuous exposure to risk factors.

The treatment strategy for AC undeniably emphasizes prevention, including measures like wearing wide-brimmed hats, using caps, avoiding sun exposure, employing lip moisturizers with sun protection, and considering ointments with vitamin E. Timely initiation of AC treatment has shown efficacy in stabilizing and even reversing the clinical condition, thereby preventing its malignant evolution.

Hence, the primary aim of this study was to assess the rate of malignant transformation of AC within a specialized cancer hospital’s reference service. The garnered results hold significance in unraveling the epidemiological profile of this lesion and proposing more targeted and effective prevention strategies.

## Material and Methods

A retrospective investigation was conducted, and clinical, histopathological, and demographic information were collected. This study’s protocols garnered endorsement from the Research Ethics Committee– (registration number 47833621.7.0000.0098). The analysis was centered on patient data afflicted with actinic cheilitis (AC) and treated at the Oral and Maxillofacial Surgery Service of a reference cancer hospital over a decade-long span from 2008 to 2018. Follow-up data and interval between first diagnosis and malignant transformation was collected.

All patients who had an initial diagnosis of AC treated from 2008 to 2018 were included in the study. Cases in which the necessary information was not available were excluded from the study, as well as cases in which a malignant neoplasm was already diagnosed in the first consultation.

## Results

A comprehensive analysis was conducted on 224 medical records of patients diagnosed with AC at the a reference cancer hospital’s Oral and Maxillofacial Surgery Service, spanning the years 2008 to 2018 ( Table 1). The gender distribution revealed that 72 (32.2%) patients were female, while 152 (67.8%) were male. The patients’ ages ranged from 24 to 89 years, with an average age of 65 years.

Regarding lifestyle factors, 94 (51.4%) patients were identified as smokers, and 58 (31.2%) reported alcohol consumption. Professions associated with a higher risk of developing AC, such as farming, security, driving, masonry, and postal services, were observed in 170 (87.6%) patients. Only 16 (9.6%) individuals reported daily use of sun protection, while 149 (89.9%) did not use any form of sun protection. Additionally, 22 (12.7%) patients had a history of skin cancer.

Analyzing patients who had undergone lip biopsy, 85 (38%) had not, while 86 (43%) had undergone a lip biopsy. Lesions were predominantly observed in the lower lip for 210 (94.5%) patients, with 4 (1.8%) in the upper lip, 3 (1.4%) in both lips.

The evolution time of AC varied widely, ranging from days to several years, with an average of 74 months and reports of up to 38 years. Clinical manifestations included loss of the vermilion border with dry areas, without ulcerations, and lesions were soft on palpation. Malignant cases exhibited ulcerated lesions, raised edges, and hardened lip regions.

Demographically, 223 (99.5%) patients had fair skin, and 1 (0.4%) had dark skin. Malignant transformations were noted in 27 patients (12.05%), with follow-up times ranging from 2 months to 20 years. While most cases were diagnosed at early stages, some were already advanced.

Patients diagnosed with malignancy were referred to the Head and Neck Surgery service, where detailed analyses led to diverse treatment approaches, including surgery, chemotherapy, and radiotherapy. Treatment protocols for AC were sustained post-surgery.

All patients received comprehensive education on the importance of preventive measures, including proper lip care, use of lip moisturizers with sun protection, avoidance of UV radiation accumulation, and the benefits of wearing caps and wide-brimmed hats. Emphasis was placed on the critical role of early diagnosis in stabilizing AC.

## Discussion

Similar to other Oral Potentially Malignant Disorders (OPMDs), Actinic Cheilitis (AC) exhibits a gradual and unpredicTable progression toward malignancy, influenced by various risk factors. The study meticulously tracked patients over a median period of 120 months, revealing an average malignant transformation rate of 12.05%. In comparison to OPMDs like leukoplakia, AC demonstrates a significantly higher likelihood of malignant transformation, emphasizing the critical need for preventive measures and early diagnostic guidance.

Unlike skin cancer, AC displays heightened aggressiveness, characterized by an increased incidence of metastasis. Both conditions share sun exposure as a common risk factor and are also linked to occupational factors. Astonishingly, despite this well-established correlation, a significant 89.8% of patients neglect sun protection, while 87.6% of cases reported engaging in risky occupations. This underestimation of risk, despite its visible nature, fosters disease progression and complicates timely diagnosis.

Lip cancer emerges as the most prevalent form of oral cancer, accounting for around 30% of tumors within this region (7). This is closely followed by tongue and floor of the mouth cancers. The predominant epidermoid type of lip cancer is typically preceded by AC lesions, sharing analogous epidemiological characteristics (1). It is more commonly seen in fair-skinned men exposed to frequent UV radiation (2). Alongside this, risk factors including smoking, alcoholism, viral infections, immunosuppression, and genetic predisposition can contribute to lip carcinogenesis (7,8).

A pivotal step in mitigating lip and skin cancer involves public policies advocating for the widespread use of sunscreens. Long-term monitoring holds significance, encouraging patient adherence to follow-up protocols and care regimens that, in turn, bolster prevention and early detection efforts (9). All participants in the study received comprehensive guidance regarding preventive measures and care protocols for AC lesions.

Clinical indicators such as ulceration, necrosis, friability, bleeding, and submucosal firmness are crucial signs necessitating biopsy and vigilant monitoring (10). The study unveiled an array of symptoms, including ulcerations, dryness, and whitish areas, sometimes overlooked by patients, thereby complicating their pursuit of medical intervention and early diagnosis.

Lip cancer emerges as the most prevalent malignant oral neoplasm. Survival rates vary from 95-100% for early-stage cancers to 37-67% for stages III and IV. The malignant transformation hinges on demographic traits and exposure to risk factors. Early diagnosis can yield a remarkable 90% cure rate, with a mortality range of 10-15%.

The study population comprised predominantly fair skin individuals, aligning with existing literature findings. Nevertheless, other factors like education proved relevant, with 55.4% of patients having only primary education, highlighting the impact of socio-economic backgrounds.

Smoking’s influence surfaced in the study, with a preference for the right side among smokers, mirroring prior research. Interestingly, 51.4% of participants were smokers, 67.8% were male, and 5.4% were in their fifth decade of life. These demographics emphasize the alignment of findings with existing literature and indicate the target population for prevention.

In Brazil, where agricultural and occupational sun exposure prevails, AC is prevalent among fair-skinned individuals. Early diagnosis holds the key to informed patient guidance and care. Notably, oncological interventions might lead to lasting impairments such as restricted mouth opening, possibly necessitating facial prosthetics for reconstruction.

## Figures and Tables

**Table 1:** Sample demographic profile and risk factors exposure.

Variable		N (%)
Sex	Female	72 (32.1)
	Male	152 (67.9)
Tobacco	Yes	94 (42)
	Not	88 (39.3)
	Not informed	42 (18.7)
Alcohol consumption	Yes	58 (25.9)
	Not	127 (56.7)
	Not informed	39 (17.4)
Solar Protection	Yes	16 (7.2)
	Not	149 (66.5)
	Not informed	59 (26.3)
History of non-melanoma skin cancer	Yes	20 (8.9)
	Not	150 (67.0)
	Not informed	54 (24.1)
Occupational risk	Yes	170 (75.9)
	Not	23 (10.3)
	Not informed	31 (13.8)
MalignantTransformation	Yes	27 (12.05)

## Data Availability

The datasets used and/or analyzed during the current study are available from the corresponding author.
